# Structure of the Lipooligosaccharide from the Deep-Sea Marine Bacterium *Idiomarina zobellii* KMM 231^T^, Isolated at a Depth of 4000 Meters

**DOI:** 10.3390/md20110700

**Published:** 2022-11-09

**Authors:** Maxim S. Kokoulin, Pavel S. Dmitrenok, Lyudmila A. Romanenko

**Affiliations:** G.B. Elyakov Pacific Institute of Bioorganic Chemistry, Far Eastern Branch, Russian Academy of Sciences, 159/2, Prospect 100 let Vladivostoku, Vladivostok 690022, Russia

**Keywords:** marine bacteria, deep-sea, *Idiomarina zobellii*, lipooligosaccharide, lipid A, NMR spectroscopy, MALDI mass spectrometry

## Abstract

The structural characterization of lipopolysaccharides has critical implications for some biomedical applications, and marine bacteria are an inimitable source of new glyco-structures potentially usable in medicinal chemistry. On the other hand, lipopolysaccharides of marine Gram-negative bacteria present certain structural features that can help the understanding of the adaptation processes. The deep-sea marine Gram-negative bacterium *Idiomarina zobellii* KMM 231^T^, isolated from a seawater sample taken at a depth of 4000 m, represents an engaging microorganism to investigate in terms of its cell wall components. Here, we report the structural study of the R-type lipopolysaccharide isolated from *I. zobellii* KMM 231^T^ that was achieved through a multidisciplinary approach comprising chemical analyses, NMR spectroscopy, and MALDI mass spectrometry. The lipooligosaccharide turned out to be characterized by a novel and unique pentasaccharide skeleton containing a very short mono-phosphorylated core region and comprising terminal neuraminic acid. The lipid A was revealed to be composed of a classical disaccharide backbone decorated by two phosphate groups and acylated by i13:0(3-OH) in amide linkage, i11:0 (3-OH) as primary ester-linked fatty acids, and i11:0 as a secondary acyl chain.

## 1. Introduction

The deep sea is an intricate ecosystem with an enormous variety of life forms, including an extensive fraction of bacterial biomass. Life in most deep-sea habitats is characterized by extreme abiotic conditions encompassing high salinity, low temperature, high hydrostatic pressure, absence of sunlight, and low input of organic matter [1,2,3]. Deep-sea marine bacteria employ a variety of adaptive strategies to survive and proliferate in a habitat that is destructive to other microorganisms. The capacity of marine bacteria to face extreme environmental stresses is predominantly determined by the stability of their cell membranes, which as a rule is provided by variations in their lipid composition. The changes in the membrane lipids are primarily observed for fatty acid composition and are apparently due to the pressure or temperature effects on the microorganisms [4,5,6,7]. In addition, as adopted strategies to survive, many deep-sea bacteria can produce extracellular polysaccharides (EPSs). Bacterial EPSs can be secreted into the extracellular medium or attached to the cell wall (capsular polysaccharides, CPSs). Both serve diverse functions, such as the formation of a microenvironment that promotes attachment to surfaces, the capture of nutrients, and protection against stressors of the surrounding environment [8,9].

As a rule, Gram-negative bacteria are externally covered by another type of glycoconjugate—lipopolysaccharides (LPS), which represent the main components of the external leaflet of the outer membrane. This lipoglycan (generally) has a three-domain structural architecture and is composed of lipid A (a hydrophobic part that anchors the LPS molecule in the outer membrane), the core oligosaccharide, which is connected to lipid A through the 3-deoxy-D-*manno*-oct-2-ulosonic acid (Kdo, sugar marker of Gram-negative bacteria) and may be substituted by the O-polysaccharide (OPS) [10]. From the chemical point of view, lipid A has a conserved structure and is commonly made up of a *bis*-phosphorylated D-glucosamine disaccharide backbone substituted with several ester- and amide-linked fatty acyl side chains. These fatty acyl residues are peculiar because they bear a 3-OH group which, in turn, can be esterified by secondary fatty acids. The lipid A part is inherently heterogeneous due to the different degrees of acylation and phosphorylation, the distribution, and the type of acyl chains. The core region consists of outer core sugars and a relatively conserved inner core, composed mainly of heptose and Kdo residues. The OPS is usually built of oligomeric repeating units (from two to eight monosaccharide residues) and, being the most exposed part, is in immediate interaction with host cells in the case of symbiotic and pathogenic bacteria [10]. LPS can be classified as smooth or rough depending on the completeness of the carbohydrate portion: smooth-type LPS (S-LPS) is built up of all three above parts, whereas rough-type LPS (R-LPS) does not contain OPS moiety. The chemical structure of LPS, especially lipid A and core oligosaccharide, is critical for outer membrane integrity, flexibility, and fluidity, as well as for normal physiology and the growth of microorganisms [10].

On the other hand, the LPS is classified as a microbe-associated molecular pattern, and it can trigger the innate immune response when recognized by receptors termed pattern recognition receptors [11]. In mammals, LPS is recognized by the Toll-like Receptor 4/Myeloid differentiation factor-2 (TLR4/MD-2) receptor complex [12,13]. The endotoxic center of LPS lies in the lipid A moiety (also influenced by the core oligosaccharide), which specifically binds to the TLR4/MD-2 complex and activates the production of pro-inflammatory cytokines in different ways, depending on its structure. The immunological properties of LPS range from strong activation (generally referred to as agonism) to only weak or no immunostimulatory effect. In contrast, LPS molecules that are ineffective in activating the TLR4-mediated signaling while retaining the ability to bind the receptor and prevent the binding of agonistic LPS are defined as antagonists [12]. Given this premise, the study of novel LPS structures, which might possess inhibitory action towards TLR4/MD-2 mediated release of pro-inflammatory mediators by agonistic LPS, is considered of high relevance. Being continuously exposed to environmental stress factors that can impact their general structure, LPSs isolated from deep-sea bacteria often show unique chemical features that influence their biological effects on the host immune system [14,15]. Considering that the bacterial biodiversity of deep-sea ecosystems is relatively unexplored, it is reasonable to hypothesize that the study of new natural LPSs may provide options for novel pharmaceuticals [14,15,16].

In light of this, here, we report the complete structural characterization of the R-LPS molecule from *Idiomarina zobellii* KMM 231^T^, isolated from a seawater sample taken at a depth of 4000 m from the northwestern Pacific Ocean. Currently, the genus *Idiomarina* comprises 14 validly described species (List of prokaryotic names with standing in nomenclature at https://lpsn.dsmz.de/genus/psychrobacter, accessed on 19 October 2022 [17]). The glycopolymers from *Idiomarina* spp. have been barely described [18,19], and information on core oligosaccharide and lipid A structures, except fatty acid composition [20], is absent from the literature. The primary structure of lipooligosaccharide (LOS) was established by a combination of chemical methods, 2D nuclear magnetic resonance (NMR), and matrix-assisted laser desorption ionization (MALDI) mass spectrometry (MS), performed on the whole macromolecule and its completely deacylated product. The structure detected was identified as a unique, highly negatively charged, deep-rough LPS in which a trisaccharide subunit is connected to a typical lipid A glucosamine backbone.

## 2. Results

### 2.1. LOS Extraction, Purification, and Chemical Analysis

**Figure 1:**
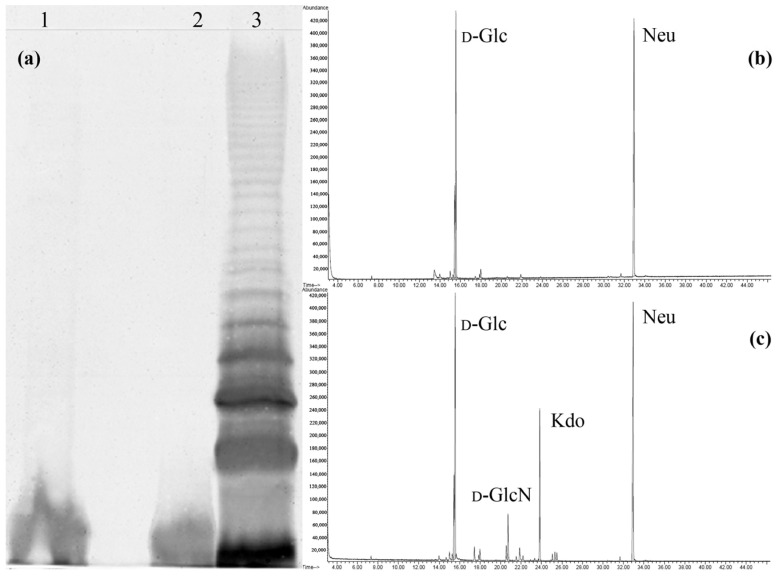
(**a**) Silver-stained electrophoregram of the LOS from *I. zobellii* KMM 231^T^ (lanes 1 and 2) and LPS *Escherichia coli* O111:B5 (lane 3); (**b**) Gas chromatography profile of the acetylated methyl glycosides derived from the native LOS from *I. zobellii* KMM 231^T^ and (**c**) after HF treatment.

Dried cells of *I. zobellii* KMM 231^T^ were extracted by the phenol/chloroform/petroleum ether (PCP) method [21] and subjected to enzymatic digestion followed by dialysis to remove cell contaminants. The silver-stained sodium dodecyl sulfate–polyacrylamide gel electrophoresis (SDS-PAGE) experiment (Figure 1a) showed the presence of only bands with a low molecular mass at the bottom of the gel, typical of R-LPS (LOS). An aliquot of pure LOS was subjected to a compositional analysis. In particular, the fatty acid analysis as methyl esters revealed mainly the occurrence of 3-hydroxyisotridecanoic [iC13:0(3-OH)], 3-hydroxyisoundecanoic [iC11:0(3-OH)], and isoundecanoic (iC11:0) acids (the stereochemistry was not determined). A minor amount of 3-hydroxydodecanoic [C12:0(3-OH)], 3-hydroxydecanoic [C10:0(3-OH)], isotridecanoic (iC13:0), and decanoic (10:0) was also detected. In turn, the results obtained from monosaccharide analysis as the acetylated methyl and (*S*)-2-butyl glycosides suggested the presence of D-glucose (D-Glc) and 5-amino-3,5-dideoxy-D-*glycero*-D-*galacto*-non-2-ulosonic acid (Neu, Figure 1b). The sugar analysis accomplished after HF treatment revealed the additional presence of 2-amino-2-deoxy-D-glucose (D-GlcN) and Kdo, indicating their phosphorylation (Figure 1c).

### 2.2. Matrix-Assisted Laser Desorption Ionization Time of Flight Mass Spectrometry (MALDI-TOF MS) Analysis of LOS

**Figure 2:**
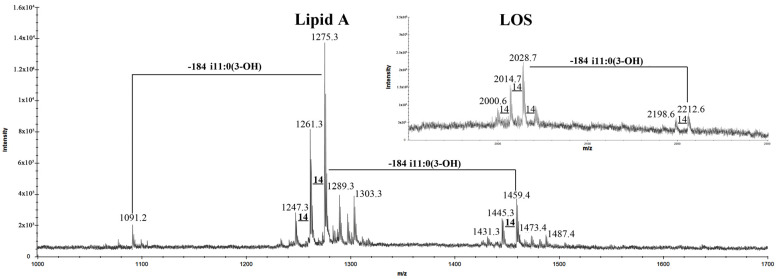
Negative-ion MALDI-TOF mass spectrum of the LOS from *I. zobellii* KMM 231^T^ recorded in reflector mode.

The LOS was analyzed by negative-ion MALDI-TOF MS, and the spectrum revealed the presence of clusters of ions related to the LOS and lipid A molecules (Figure 2). At high molecular mass values, based on compositional analysis, the signal at *m*/*z* 2028.7 was attributed to a tetra-acylated LOS molecule with the following composition: NeuAcGlcKdoPGlcN_2_P_2_[i13:0(3OH)]_2_[i11:0(3OH)](i11:0) (calculated [M − H]^−^ = 2028.9 Da). The spectrum also showed the presence of a signal at *m*/*z* 2212.6, which was assigned to penta-acylated isoform (calculated [M − H]^−^ = 2213.0 Da), which differs from the previous one by the presence of an additional i11:0(3OH) residue. At lower molecular masses, signals attributable to lipid A were visible. The ion peaks at *m*/*z* 1459.4, 1275.3, and 1091.2 (calculated [M − H ^−^ = 1459.9, 1275.7, and 1091.6 Da, respectively) were assigned to di-phosphorylated penta-, tetra- and tri-acylated lipid A molecules, respectively, differing for a [i11:0(3-OH)] unit (Figure 2). To establish the structure of the core oligosaccharide, a full deacylation of the LOS, followed by gel-permeation chromatography, was performed, and the obtained oligosaccharide fraction (OS) was studied by NMR spectroscopy.

### 2.3. NMR Spectroscopy Structural Elucidation of the R-LPS Core Oligosaccharide

**Figure 3:**
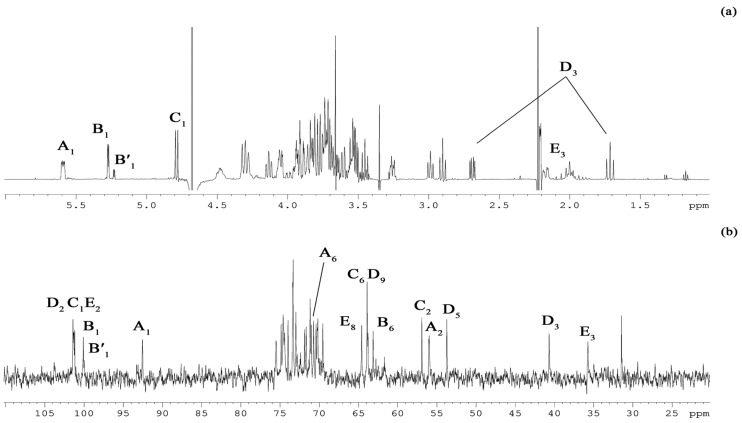
^1^H (**a**) and ^13^C NMR (**b**) spectra of the OS. Numerals refer to carbons in sugar residues denoted by capital letters as described in Table 1.

The sugar analysis of the OS confirmed the presence of the monosaccharide residues found in the LOS (Figure 1b,c). The ^1^H NMR spectrum of the OS (Figure 3a and Table 1) showed, inter alia, signals for three major anomeric protons at *δ*_H_ 5.59 (^3^*J*_H1–H2_ 3.1 Hz, H-1 of residue **A**), 5.27 (^3^*J*_H1–H2_ 3.6 Hz, H-1 of residue **B**), and 4.79 (^3^*J*_H1–H2_ 8.5 Hz, H-1 of residue **C**). 

In addition, the signals at *δ*_H_ 1.71/2.69 and 2.00/2.17 were identified as two pairs of H-3 methylene protons belonging to the Neu (**D**) and Kdo (**E**) residues. The ^13^C NMR spectrum of the OS (Figure 3b and Table 1) contained, inter alia, signals for five major carbons in the anomeric region at *δ*_C_ 101.4 (C-2 of residue **D**), 101.3 (C-1 of residue **C**), 101.2 (C-2 of residue **E**), 100.1 (C-1 of residue **B**), and 92.5 (C-1 of residue **A**); three carbons linked to nitrogen at *δ*_C_ 56.9 (C-2 of residue **C**), 56.0 (C-2 of residue **A**), and 53.7 (C-5 of residue **D**); five hydroxymethyl carbons at *δ*_C_ 63.1 (C-6 of residue **B**), 63.8 (C-9 of residue **D**), 63.9 (C-6 of residue **C**), 64.6 (C-8 of residue **E**), and 70.7 (C-6 of residue **A**, data of the DEPT-135 experiment); and two methylene groups at *δ*_C_ 40.7 (C-3 of residue **D**) and 35.7 (C-3 of residue **E**). Finally, the ^31^P spetrum (data not shown) revealed three different monophosphate ester groups with chemical shifts at *δ*_P_ 2.46, 3.25, and 4.17. The OS product was fully characterized (Table 1) by the set of homo- and hetero-nuclear 2D NMR experiments (^1^H,^1^H COSY, ^1^H,^1^H TOCSY, ^1^H,^1^H ROESY, ^1^H,^13^C HSQC, ^1^H, ^13^C TOCSY-HSQC, ^1^H,^13^C HMBC, and ^1^H,^31^P HMBC).

**Figure 4:**
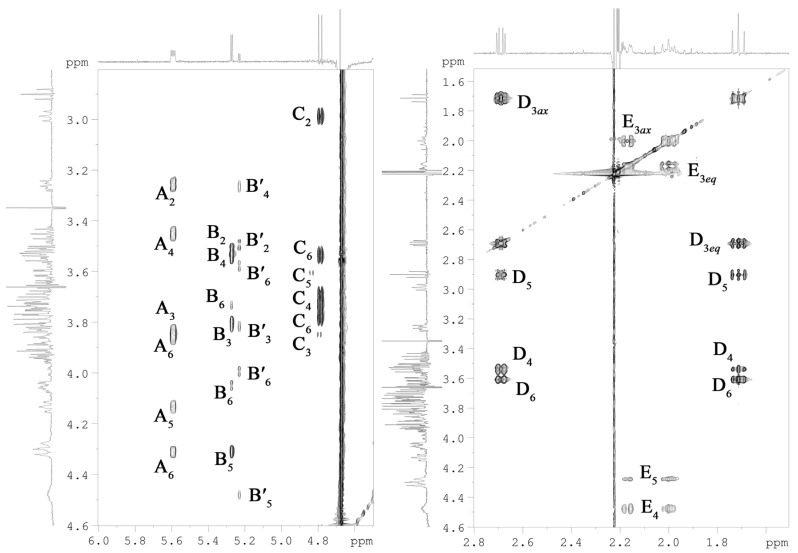
Fragments of ^1^H,^1^H TOCSY spectrum of the OS. Numerals refer to carbons in sugar residues denoted by capital letters as described in Table 1.

**Figure 5:**
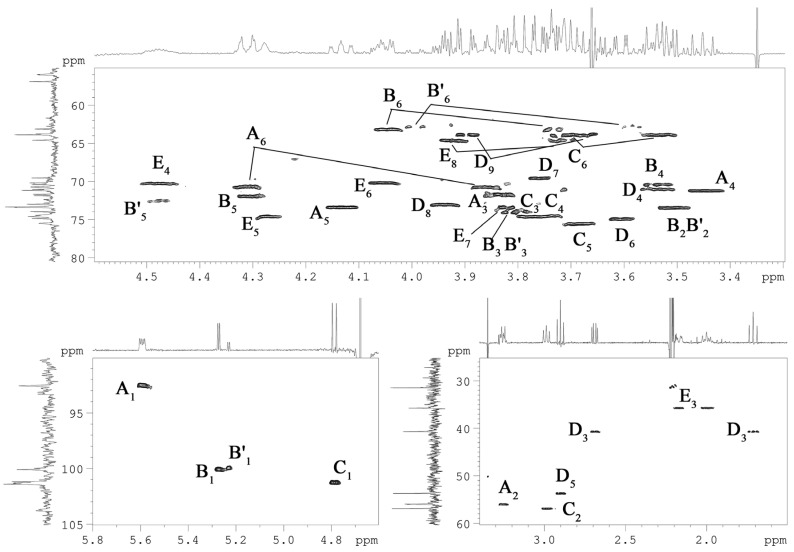
^1^H, ^13^C HSQC spectrum of the OS. Numerals refer to carbons in sugar residues denoted by capital letters, as described in Table 1.

**Figure 6:**
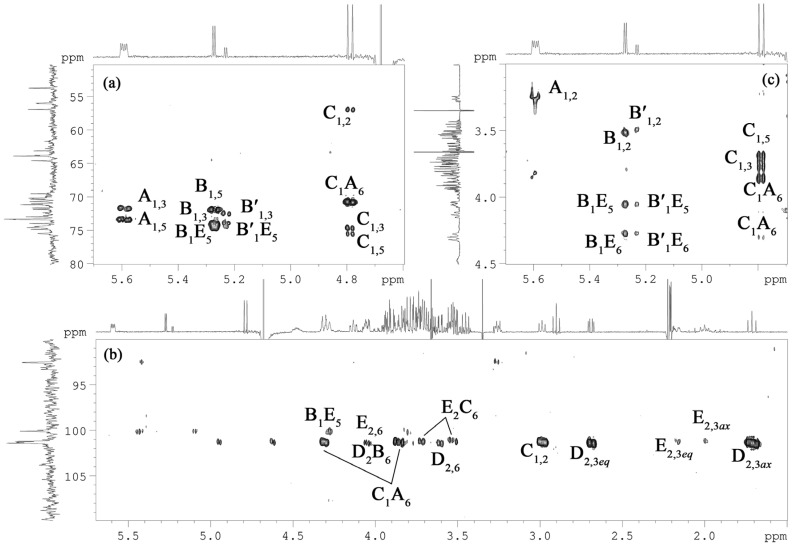
Fragments of ^1^H, ^13^C HMBC (**a**,**b**), and ^1^H,^1^H ROESY (**c**) spectra of the OS. Numerals refer to carbons in sugar residues denoted by capital letters as described in Table 1.

Briefly, each spin system could be assigned by analysis of the ^1^H,^1^H COSY, and ^1^H,^1^H TOCSY (Figure 4) spectra, with the ^1^H, ^13^C HSQC (Figure 5), ^1^H, ^13^C TOCSY-HSQC, and ^1^H,^13^C HMBC (Figure 6a,b) spectra that led to the identification of each carbon atom. The anomeric configuration of each aldose residue was assigned based on the ^3^*J*_H1-H2_ coupling constants and confirmed by the *intra*-residual nuclear Overhauser effect (NOE) contacts observed in the ^1^H,^1^H ROESY (Figure 6c) spectrum. The first two residues (**A** and **B**) were identified as α-configured, whereas residue **C** was β-configured. The ^3^*J*_H,H_ ring coupling constant values (8–10 Hz) allowed the identification of the relative *gluco*-configuration of hydroxyl groups within each aldose unit. According to the ^13^C chemical shift values (Table 1) and the long-range correlations between H-1 and C-5 in the ^1^H, ^13^C HMBC spectrum (for the Kdo*p* and Neu*p* residues between C-2 and H-6), and all the monosaccharide residues were in pyranose form. The ^1^H, ^31^P HMBC experiment was used to establish the location of the phosphate groups. In detail, spin systems **A** and **C** were assigned to the proximal and distal D-Glc*p*N residues of the lipid A disaccharide backbone based on their H-2/C-2 correlations at *δ*_H_*/δ*_C_ 3.26/56.0 and 2.99/56.9 in the ^1^H,^13^C HSQC spectrum (Figure 5), respectively. Both D-Glc*p*N units were phosphorylated, as evidenced by the observation of cross-peaks between H-1 of residue **A**, H-4 of residue **C,** and corresponding phosphate groups at δ_H_/δ_P_ 5.59/2.46 and 3.73/4.17, respectively (data of the ^1^H, ^31^P HMBC experiment). The observation of the *inter*-residue NOE cross-peaks **C** H-1/**A** H-6 at δ_H_/δ_H_ 4.79/4.31, 3.86 (data of the ^1^H,^1^H ROESY spectrum), and long-range scalar correlation **C** H-1/**A** C-6 at δ_H_/δ_C_ 4.79/70.7 (data of the ^1^H,^13^C HMBC spectrum) validated the assignment of residues **A** and **C** to the lipid A moiety. Spin system **B** and minor spin system **B′** were attributed to α-D-Glc*p* residues. 

Residue **E** was identified as the Kdo*p* unit and assigned starting from the two diastereotopic protons resonating at δ_H_ 2.17 and 2.00. In the ^1^H,^1^H TOCSY spectrum (Figure 4), both protons were correlated to signals at δ_H_ 4.48 and 4.28, which are assigned to H-4 and H-5, respectively. The downfield shift of the C-5 signal at δ_C_ 74.5, compared to the value for the unsubstituted monosaccharide [22], suggested glycosylation of Kdo*p* residue at the *O*-5 position. Moreover, the H-4 and C-4 chemical shifts, downfield shifted at δ_H_ 4.48 and δ_C_ 70.3, respectively, indicated the phosphorylation of Kdo*p* residue at this position. This finding was confirmed by the ^1^H,^31^P-HMBC spectrum, which showed the correlation between H-4 and phosphate group at δ_H_/δ_P_ 4.28/3.25. The α-configuration of Kdo*p* residue was suggested based on the difference (0.17 ppm) between H-3*eq* and H-3*ax* chemical shifts and confirmed by the C-6 chemical shift at δ_C_ 70.2 (compared to the published data for methyl 3-deoxy-α- and -β-manno-oct-2-ulopyranosonate [22]. Finally, the residue **D** was identified as the Neu*p*. The spin system was assigned starting from its diastereotopic H-3 methylene protons resonating at δ_H_ 1.71 and 2.69 (H-3*ax* and H-3*eq*, respectively). The coupling constant values of ^3^*J*_3ax,4_, ^3^*J*_4,5_ and ^3^*J*_5,6_ of ~11 Hz, ^3^*J*_6,7_ of ~2 Hz, ^3^*J*_7,8_ of ~9 Hz, and ^3^*J*_8,9ax_ of ~12 Hz were in agreement with the D-*glycero*-D-*galacto*-configuration of nonulosonic acid [23]. The ^1^H,^13^C HSQC spectrum showed the correlation of H-5 at δ_H_ 2.90 with the nitrogen-bearing carbon at δ_C_ 53.7, indicating the presence of the amino group at position C-5. The chemical shift of H-3*eq* of δ_H_ 2.69 corresponded to the α-configuration of the anomeric center (axial carboxyl group).

The sequence of the monosaccharide residues was deduced through the *inter*-residues NOE contacts and long-range scalar correlations in the ^1^H,^13^C HMBC spectrum (Figure 6). Starting from the lipid A disaccharide backbone composed of residues **A** and **C**, this latter was found to be substituted at the canonical *O*-6 position by the Kdo*p* residue (**E**). It was indicated by the observation of the weak downfield shift of the residue **C** C-6 signal, typical of ketose glycosylation, and by the long-range correlation C-2 **E**/H-6 **C** at δ_C_/δ_H_ 101.2/3.71, 3.53 in the ^1^H,^13^C HMBC spectrum. The Kdo*p* residue (**E**) was, in turn, substituted at *O*-5 by the α-D-Glc*p* residue (**B**), as evidenced by the correlations H-1 **B**/H-5 **E** at δ_H_/δ_H_ 5.27/4.28 and H-1 **B**/C-5 **E** at δ_H_/δ_C_ 5.27/74.6 in the ^1^H,^1^H ROESY, and ^1^H,^13^C HMBC spectra, respectively. In addition, the α-D-Glc*p* residue (**B**) was further glycosylated by the α-Neu*p* unit (**D**) at its *O*-6, as attested by the scalar correlation C-2 **D**/H-6 **B** at δ_C_/δ_H_ 101.4/4.05 (^1^H,^13^C HMBC spectrum) and by the NOE contacts of axial H-3 of residue **D** with both H-6 of residue **B** at δ_H_/δ_H_ 1.71/4.05, 3.73 (data of the ^1^H,^1^H ROESY spectrum). Based on NMR chemical shifts (Table 1), minor spin system **B′** was attributed to the non-substituted α-D-Glc*p* residue (the absence of α-Neu*p* residue). 

Thus, the primary structure of the OS is sketched below:

α-Neu*p*-(2→6)-α-D-Glc*p*-(1→5)-α-Kdo*p*4P-(2→6)-β-D-Glc*p*N4P-(1→6)-β-D-Glc*p*N1P
**D**        **B**        **E**        **C**        **A**

To determine the complete structure of the glycolipid portion and establish the distribution of the acyl chains on the disaccharide backbone, mild acid hydrolysis of the LOS was performed, and obtained lipid A fraction was studied by MALDI-TOF MS.

### 2.4. MALDI-TOF MS Analysis of Lipid A

**Figure 7:**
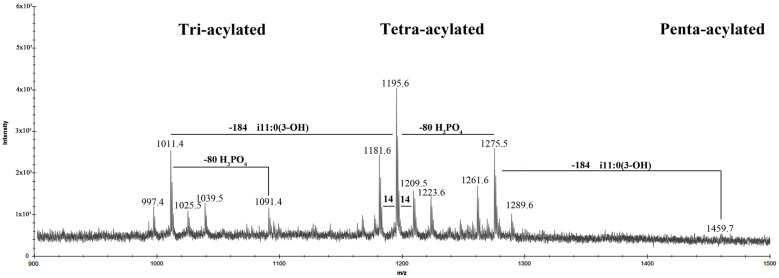
Negative ions MALDI-TOF mass spectrum of the lipid A from *I. zobellii* KMM 231^T^ recorded in reflector mode.

The negative-ion MALDI-TOF spectrum of the lipid A revealed two clear clusters of signals, corresponding to glycoforms with different patterns of fatty acids and phosphate content (Figure 7). In addition, the central peaks were characterized by the presence of mass differences of 14 Da (–CH_2_– unit), which are diagnostic for lipid A species differing in the length of acyl chains and corresponded to the compositional analysis data. The ion peaks at *m*/*z* 1275.5, 1195.6, 1091.4, and 1011.4 corresponded to *bis*- and mono-phosphorylated tetra- and tri-acylated glycoforms, respectively. In detail, based on the fatty acid composition, the ion at *m*/*z* 1275.5 was attributed to a *bis*-phosphorylated tetra-acylated lipid A species carrying two units of i13:0(3-OH), one i11:0(3-OH) and one i11:0. The corresponding mono-phosphorylated lipid A form was assigned to the peak at *m*/*z* 1195.6. A *bis*-phosphorylated tri-acylated lipid A species lacking one i11:0(3-OH) residue was attributed to the ion at *m*/*z* 1091.4, whose mono-phosphorylated form was assigned to the ion at *m*/*z* 1011.4, respectively. The minor peak at *m*/*z* 1459.7 was related to the di-phosphorylated penta-acylated species with an additional i11:0(3OH) unit.

**Figure 8:**
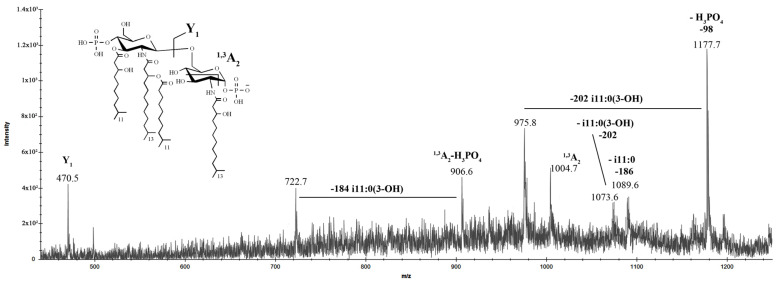
MALDI tandem mass spectrometry (MS/MS) analysis of the tetra-acylated lipid A species at *m/z* 1275.5 from *I. zobellii* KMM 231^T^. Fragment assignments are indicated. The proposed structure for the tetra-acylated lipid A species is reported in the inset.

The negative-ion MS/MS analysis was conducted to reveal the location of the lipid A acyl moieties with respect to the glucosamine disaccharide backbone. To describe *inter*- and *intra*-ring fragmentations, the nomenclature introduced by Costello and Vath [24] was used. In detail, the MS/MS spectrum of the precursor ion at *m*/z 1275.5 (Figure 8) showed an intense peak at *m*/*z* 1177.7, which corresponded to the elimination of a phosphate group. 

The ion peaks at *m*/*z* 1089.6 and 1073.6 were assigned to the fragments that originated from the loss of i11:0 and i11:0(3-OH) fatty acids, respectively. The peak at *m*/*z* 975.8 was attributed to the fragment devoid of the phosphate group and the i11:0(3-OH) unit. The absence in the spectrum of peaks corresponding to the elimination of i13:0(3-OH) residues indicated that these fatty acids were bound to amino groups. Moreover, the absence of ions related to the loss of whole *O*-acyloxyacyl moiety suggested that the secondary i11:0 acyl chain was attached to the amide-linked fatty acid. Ion Y_1_ at *m*/*z* 470.5, resulting from the cleavage of the glycosidic bond of the glucosamine backbone, demonstrated that the proximal α-D-Glc*p*N^I^ was mono-acylated [i13:0(3-OH)] whereas distal β-D-Glc*p*N^II^ was tri-acylated (i13:0(3-OH), i11:0(3-OH), and i11:0). In parallel, the occurrence of the ion ^1,3^A_2_ at *m*/*z* 1004.7, originating from the sugar cross-ring cleavage, was fundamental to confirm the nature of the fatty acids that decorated the distal β-D-GlcpN^II^. In support of this, the ions originating from the sugar ring fragmentation ^1,3^A_2_, in addition to the loss of the phosphate group and i11:0(3-OH) acyl moiety, were assigned to the peaks at *m*/*z* 906.6 and 722.7, respectively. 

**Figure 9:**
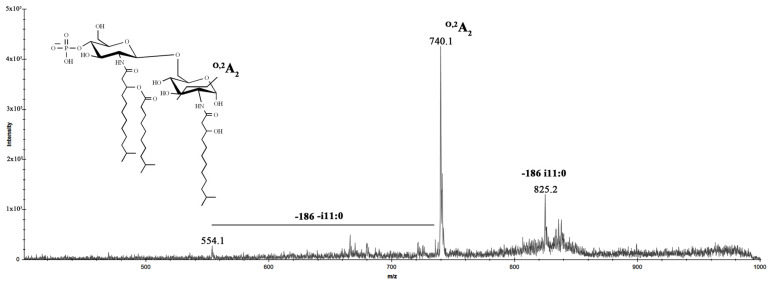
MALDI MS/MS analysis of the tri-acylated lipid A species at *m*/*z* 1011.4 from *I. zobellii* KMM 231^T^. Fragment assignments are indicated. The proposed structure for the tri-acylated lipid A species is reported in the inset.

The negative-ion MS/MS spectrum of the precursor ion at *m*/*z* 1011.4 (Figure 9), due to mono-phosphorylated lipid A species bearing two i13:0(3-OH) and one i11:0 unit, showed an ion at *m*/*z* 825.2 corresponding to the lipid A fragment missing i11:0 residue. 

Moreover, it demonstrated an intensive peak at *m*/*z* 740.1 corresponding to the ^O,2^A_2_ ion; this fragment confirmed that i13:0(3-*O*-i11:0) residue was attached to distal β-D-GlcpN^II^. An aliquot of lipid A was subjected to hydrolysis with ammonium hydroxide to verify the position of the secondary fatty acid. The negative-ion MALDI mass spectrum of the modified lipid A did not differ from the spectrum of the native one (data not shown), except for the increase in the intensity of the ion peaks at *m*/*z* 1091 and 1011, corresponding to the mentioned tri-acylated lipid A species.

**Figure 10:**
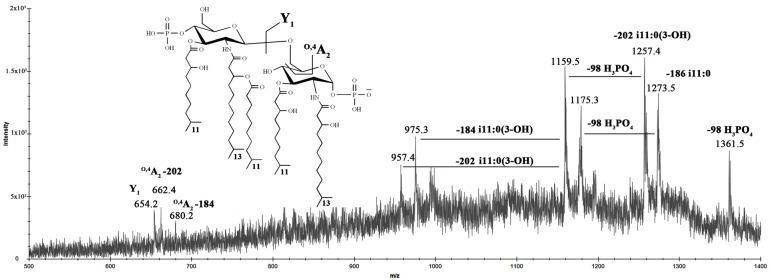
MALDI MS/MS analysis of the penta-acylated lipid A species at *m*/*z* 1459.4 from *I. zobellii* KMM 231^T^. Fragment assignments are indicated. The proposed structure for the penta-acylated lipid A species is reported in the inset.

Finally, to establish the position of the i11:0(3-OH) fatty acid on the penta-acylated form of lipid A found from the MALDI spectrum of the LOS, MS/MS analysis of the precursor ion at *m*/*z* 1459.4 was conducted (Figure 10).

MS/MS spectrum of this ion also provided a set of successive losses of a phosphate group and acyl chains. The ion Y_1_ at *m*/*z* 654.2 demonstrated that i11:0(3-OH) residue attached to proximal D-GlcN^I^. Moreover, the absence of ions ^1,3^A_2_ or ^O,2^A_2_, which usually occur when the *O*-3 hydroxyl group of the reducing D-GlcN^I^ was not substituted [25], suggested that the additional i11:0(3-OH) unit was at this position. 

**Figure 11:**
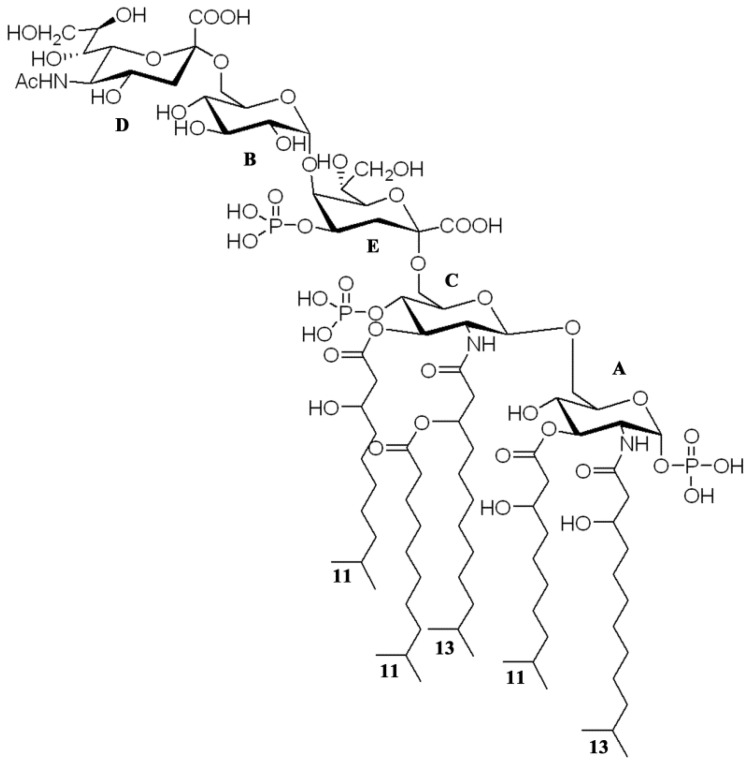
Proposed structure of the LOS from *I. zobellii* KMM 231^T^.

Thus, taking the results of the compositional, NMR spectroscopy, MS, and MS/MS analysis of the LOS, OS, lipid A, and the ammonium-hydroxide-treated product, respectively, it became possible to determine the *I. zobellii* KMM 231^T^ LOS structure; the structure with the highest degree of acylation is shown in Figure 11.

## 3. Discussion

Bacterial adaptations to harsh environmental conditions include the chemical modification of the membrane system to reinforce the overall cell envelope and resist the parameters of extreme habitats. Indeed, the structure of the LPSs from marine and other extremophilic bacteria often presents unusual chemical characteristics that help the microorganism maintain membrane integrity [14,26].

In this paper, the characterization of the structure of the LOS isolated from the deep-sea bacterium *I. zobellii* KMM 231^T^ is reported. Briefly, the LOS turned out to be characterized by a novel and unique pentasaccharide skeleton comprising a very short mono-phosphorylated core region and containing terminal Neu residue. The lipid A was revealed to be composed of a D-GlcN disaccharide backbone decorated by two phosphate groups and acylated by i13:0(3-OH) in amide linkage, i11:0 (3-OH) as primary ester-linked fatty acids, and i11:0 as a secondary acyl chain. Neu residues have been previously found in the outer core region of *Campylobacter jejuni* and *Helicobacter pylori* that can express either Lewis antigens or resemble structural similarities with glycosphingolipids of the ganglioside group [27]. Among marine bacteria, terminal Neu residue was found in the LOS from *Loktanella rosea* KMM 6003^T^ [28]. Unlike in the case of pathogens, the presence of Neu is an attempt to evade the host’s immune system, and its role in marine microorganisms is currently unknown.

It is considered that a short, negatively-charged oligosaccharide moiety confers to the LOS molecule the ability to vigorously interact with divalent cations that, in turn, gives rigidity and resistance to the outer membrane and whole bacterial cell. On the other hand, various anionic functional groups on the LOS could act as a buffering system, regulating the pH on the external membrane surface and protecting the bacterium from salinity conditions. In addition, the presence of short, branched acyl chains composing the lipid A moiety can be considered a direct consequence of the necessity of microorganisms to reach a required outer membrane fluidity, thus protecting the bacterial cell from low temperatures and high-pressure conditions [14]. Together, these facts may explain why marine bacteria can survive in the deep-sea environment.

Depending on the nature and distribution of the acyl chains, the lipid A molecules may induce agonistic or antagonistic activity after interaction with the TLR4/MD-2 receptor complex. It is widely known that the hexa-acylated *bis*-phosphorylated lipid A from *E. coli* exhibits a high agonistic activity binding to TLR4/MD-2 complex, triggering the downstream signaling. Subtle chemical modifications in certain parts of the lipid A structure are responsible for the innate immune response. The classical antagonists are the *bis*- and mono-phosphorylated tetra- and penta-acylated lipids A [10,12]. Given the elucidated structure, the immunomodulatory activity of the LOS from *I. zobellii* KMM 231^T^ is also worth being studied since it can potentially provide antagonistic properties.

## 4. Materials and Methods

### 4.1. Isolation and Purification of the LOS

*I. zobellii* KMM 231^T^ was obtained from the Collection of Marine Microorganisms (KMM) of the G.B. Elyakov Pacific Institute of Bioorganic Chemistry, Far Eastern Branch of the Russian Academy of Sciences (Vladivostok, Russia). The bacteria were cultivated as previously described [18]. The crude LOS (54 mg) was extracted from dried cells (5.63 g) by the PCP method [21]. The sample was washed with a mixture of chloroform-methanol (1:2, *v*/*v*) to remove phospholipids and was resuspended in 5 mL of digestion buffer containing tris(hydroxymethyl)aminomethane/ethylenediaminetetraacetic acid (TRIS/EDTA, 0.01 M/0.001 M) and 0.01 M MgCl_2_. After addition of 1 mg of proteinase K (Sigma-Aldrich, Saint Louis, MO, USA), the solution was incubated for 2 h at 60 °C. After dialysis against distilled water (MWCO 8000 Da), it was lyophilized to give pure LOS (43.8 mg).

### 4.2. SDS-PAGE Analysis of the LOS

Electrophoresis of the LOS sample was performed in 15% (*w*/*v*) polyacrylamide gel according to Laemmli protocol [29], and bands were visualized after silver nitrate staining [30]. LPS from *E. coli* O111:B4 (Sigma-Aldrich, USA) was used as a LPS standard.

### 4.3. Chemical Analysis of the LOS

Monosaccharides were analyzed as the acetylated methyl glycosides, using appropriate authentic samples as reference. The LOS sample (1 mg) was subjected to a methanolysis reaction with acetylchloride in methanol (1.25 M, 1 mL) at 80 °C for 16 h. The methanol was extracted three times with hexane to separate the fatty acid methyl esters (FAME) from the *O*-methyl glycosides. The hexanic phase was dried and analyzed by GC-MS. The methanolic phase was dried, and then, the *O*-methyl glycosides were acetylated with acetic anhydride in pyridine at ambient temperature for 16 h. The absolute configurations of sugar residues were determined by GC-MS of the acetylated (*S*)-2-octyl glycosides as described [31]. All the derivatives were analyzed using a Hewlett Packard 5890 chromatograph (Conquer Scientific, Poway, CA, USA) equipped with a Hewlett Packard 5973 mass spectrometer (USA) and a HP-5MS capillary column. Acetylated methyl glycosides (AMG) were analyzed using the following temperature program: 150 °C for 3 min, 150 °C → 250 °C at 3 °C/min^−1^, and 250 °C for 10 min. For the acetylated (*S*)-2-octyl glycosides, the analysis was performed at 160 °C for 3 min, 160 °C → 290 °C at 3 °C/ min^−1^, and 290 °C for 10 min. The following temperature program was employed for the FAME analysis: 140 °C for 3 min, 140 °C → 280 °C at 3 °C/min^−1^, and 280 °C for 10 min. To obtain dephosphorylated LOS, the sample (1 mg) was treated with aq 48% HF (0.1 mL, 4 °C, 16 h). HF was evaporated and modified LOS was analyzed as described above.

### 4.4. Deacylation of the LOS

The LOS sample (20 mg) was treated with *aq* 12.5% NH_4_OH (37 °C, 16 h), and the de-*O*-acylated LOS was lyophilized. The de-*O*-acylated LOS was subjected to a reaction with 4 M KOH (1.0 mL) for 16 h at 120 °C. The reaction mixture was neutralized with 2 M HCl (until pH ~6) and extracted with CHCl_3_ three times. The aqueous layer was desalted on the Toyopearl HW-40 column (120 × 1.5 cm, Tosoh Bioscience, Tokyo, Japan) eluted with *aq* 0.1% AcOH, yielding the OS (3.9 mg). Elution was monitored with a differential refractometer (Knauer, Griesheim, Germany).

### 4.5. Isolation of the Lipid A

In order to study the lipid A structure, an aliquot (10 mg) of pure LOS was treated with acetate buffer (pH 4.4) for 3 h at 100 °C. A mixture of chloroform and methanol was added to the hydrolysate to reach a chloroform/methanol/hydrolysate 2:2:1.8 (*v*/*v*/*v*) ratio. The mixture was then shaken and centrifuged. The organic phase, containing the lipid A fraction, was collected and washed with the water phase of a freshly prepared Bligh/Dyer mixture (chloroform/methanol/water, 2:2:1.8). The chloroform phase was dried and analysed by MALDI MS. An aliquot of the lipid A was also treated with *aq* 10% NH_4_OH as described [32], and analysed by MALDI MS.

### 4.6. NMR Spectroscopy and MALDI-TOF MS Analysis

^1^H and ^13^C NMR spectra of the OS were recorded on a Bruker Avance-III (700.13 MHz for ^1^H and 176.04 MHz for ^13^C) spectrometer (Bruker, Karlsruhe, Germany) at 37 °C in 99.95% D_2_O using acetone (*δ*_C_ 31.45, *δ*_H_ 2.225) as the internal calibration standard. The ^1^H,^1^H-TOCSY and ^1^H,^1^H-ROESY spectra were recorded with a 180 duration of MLEV-17 spin-lock and a 300 ms mixing time, respectively. ^1^H,^13^C-HMBC and ^1^H,^31^P-HMBC were optimized for an 8 Hz long-range constant. NMR study was carried out with recommendations as described [33].

MALDI-TOF mass spectra were acquired on an ULTRAFLEX III (Bruker, Karlsruhe, Germany) mass spectrometer. A solution of trihydroxyacetophenone (THAP) in CH_3_OH/*aq* 0.1% trifluoroacetic acid (TFA)/ CH_3_CN (7:2:1, *v*/*v*/*v*) at a concentration of 75 mg/mL was used as the matrix. The LOS and lipid A samples were desalted on a Dowex 50WX8 (H+ form) and dissolved in 2-propanol/water with a 1:1 ratio and in chloroform/methanol 3:1, respectively.

## Figures and Tables

**Table 1:** ^1^H and ^13^C NMR data for the OS from the LOS of *I. zobellii* KMM 231^T^, *δ*, ppm.

Sugar Residue	H-1 C-1	H-2 C-2	H-3eq,ax C-3	H-4 C-4	H-5 C-5	H-6a,b C-6	H-7 C-7	H-8 C-8	H-9 C-9
α-D-Glc*p*N1P **A**	5.59 92.5	3.26 56.0	3.84 71.7	3.45 71.2	4.13 73.3	4.31, 3.86 70.7			
→6)-α-D-Glc*p*-(1→ **B**	5.27 100.1	3.51 73.4	3.81 74.0	3.54 70.4	4.31 71.9	4.05, 3.73 63.1			
→6)- α-D-Glc*p*-(1→ **B′**	5.23 100.0	3.49 73.4	3.82 74.0	3.26 72.4	4.31 71.9	3.99, 3.58 62.8			
→6)-α-D-Glc*p*N4P-(1→ **C**	4.79 101.3	2.99 56.9	3.79 74.6	3.73 74.4	3.69 75.5	3.71, 3.53 63.9			
α-Neu*p*-(1→ **D**	175.3	101.4	2.69, 1.71 40.7	3.54 71.0	2.90 53.7	3.61 74.9	3.76 69.5	3.94 73.0	3.90, 3.68 63.8
→5)-α-Kdo*p*4P-(1→ **E**	176.1	101.2	2.17, 2.00 35.7	4.48 70.3	4.28 74.5	4.05 70.2	3.82 73.3	3.93, 3.72 64.6	

## Data Availability

Not applicable.
